# Molecular and serological prevalence of influenza A viruses in poultry and poultry farmers in the Ashanti region, Ghana

**DOI:** 10.1080/20008686.2019.1698904

**Published:** 2019-12-14

**Authors:** Ayim-Akonor Matilda, May Juergen, Ralf Krumkamp, Harder Timm, Mertens Eva

**Affiliations:** a Department of Infectious Disease Epidemiology, Bernhard Nocht Institute for Tropical Medicine, Hamburg, Germany; b Department of Animal Health and Food safety, Council for Scientific and Industrial Research-Animal Research Institute, Accra, Ghana; c Friedrich-Loeffler-Institut, Institute for Diagnostic Virology, Insel Riems, Germany

**Keywords:** Influenza A virus, commercial chicken, farmers, Ghana

## Abstract

For an analysis of the prevalence of influenza A viruses (IAVs) circulating in chickens and their farmers in the Ashanti region, Ghana, we examined 2,400 trachea and cloaca swabs (chickens) and 102 oropharyngeal swabs (farmers) by qRT-PCR. Sera from 1,200 (chickens) and 102 (farmers) were analysed for IAV antibodies by ELISA and haemagglutination inhibition (HI). Avian influenza virus (AIV) was detected in 0.2% (n = 5) of chickens but not farmers. Virus detection was more pronounced in the cloacal (n = 4, 0.3%) than in tracheal swabs (n = 1, 0.1%). AIV antibodies were not detected in chickens. Two farmers (2.0%) tested positive to human seasonal IAV H1N1pdm09. Sixteen (15.7%) farmers tested seropositive to IAV of which 68.8% (n = 11) were due to H1N1pdm09-specific antibodies. AIV H5- or H7-specific antibodies were not detected in the farmers. Questionnaire evaluation indicated the rare usage of basic personal protective equipment by farmers when handling poultry. In light of previous outbreaks of zoonotic AIV in poultry in Ghana the open human-animal interface raises concern from a OneHealth perspective and calls for continued targeted surveillance.

## Introduction

Worldwide, influenza A viruses (IAVs) are important veterinary and public health pathogens causing substantial morbidity and mortality in varying species including humans and poultry [1,2]. The viruses are facing host restriction barriers, but interspecies transmission with variable sequelae can occur: (i) abortive infection, (ii) productive infection associated or not with clinical disease, (iii) adaptation to new host species with secondary virus transmission. For avian influenza viruses (AIVs) high pathogenic (HP) and low pathogenic (LP) phenotypes have been described and both can harbour zoonotic propensity. The impact of HPAIVs on livelihood and food security especially of low-income countries can be immense due to the highly lethal course of disease especially in gallinaceous poultry [1]. Outbreaks of LPAIVs in gallinaceous poultry do not necessarily receive control responses in contrast to HPAIV outbreaks. However, when allowed to continuously circulate in gallinaceous poultry, LPAIV of subtypes H5 and H7 can mutate to notifiable HPAIV; other subtypes may reassort with other IAVs of avian, porcine or human origin to generate strains with extended zoonotic and even human pandemic potential [3–5]. Sporadic human infections with AIVs have been reported worldwide with higher incidences among individuals in direct contact with infected poultry, contaminated poultry products and/or poultry environment [6–9]. There has been a growing interest in AIV infections in Africa following the introduction of HPAIV H5N1 in gallinaceous poultry in 2006 [1], contributing to the identification of different AIV subtypes with known and unknown zoonotic propensities in birds on the continent [5,10–14]. Simultaneously, evidence of AIV infections, exposures and death among humans in regular contact with poultry on the continent have also increased [9,15,16].

In Ghana, outbreaks of zoonotic HPAIV H5N1 (clade 2.2 and 2.3.2.1c) in poultry have been reported with no human deaths [9,17]. Studies focusing on active infection after the first outbreak (in 2007), recognised an increased risk of zoonotic transmission due to poor implementation of biosecurity and biosafety practices among poultry handlers [18–20].

The Ashanti region is the second-largest commercial poultry-producing region in Ghana. An HPAIV H5N1 outbreak was recorded in the region only during the second HPAI outbreak in the country in 2015. The region is a hub for trading live poultry and/or poultry products to other parts of the country and to neighbouring countries. Little is known about AIV infections in commercial poultry and much less of poultry handlers within the area. AIV was not detected in surveillance carried out in commercial poultry before the first H5N1 outbreak in the country, and in backyard poultry in military barracks in the region after the first outbreak [20]. We performed a cross-sectional study to determine the prevalence of IA viruses in commercial chickens and their farmers within the Ashanti region of Ghana. This will contribute to our understanding of influenza at the human-animal interface in the region and aid to develop IAV control strategies to prevent infections in poultry and humans.

## Materials and methods

### Ethics and sampling

Ethical approval was obtained from the Council for Scientific and Industrial Research (RPN 001/CSIR-IACUC/2016), Ghana, and Ärztekammer Hamburg (PV5296), Germany. Between April 2016 to February 2017 tracheal and cloacal swabs and blood samples (2 mL) were collected from 1,200 clinically healthy chickens raised exclusively in-house on 76 commercial chicken farms in the Ashanti region. An oropharyngeal swab and a blood sample (2 mL) were obtained from 102 farmers from 39 of these farms. None of the farmers had symptoms suggestive of any respiratory illness at the time of sampling. Swabs were collected into viral transport medium [21] and transported on ice to the laboratory. Questionnaires were used to collect relevant farm and farmer data.

### Laboratory analysis

RNA was isolated from swabs (QIAamp viral RNA mini kit, Germany) and tested for influenza A Matrix-specific gene by qRT-PCR [22]. All positive samples were subjected to direct subtyping of all AIV subtypes [23,24]. Additionally, human samples were tested for seasonal influenza viruses of subtypes H1 and H3 by qRT-PCR [24]. Viral isolation in embryonated chicken eggs and MDCK cells of positive samples was attempted. ELISA was used to test sera for IAV antibodies (IDEXX AI MultiS-Screen kit, chicken; Serion IgG ELISA kit, human). Hemagglutination inhibition (HI) assay was used to test ELISA positive sera for avian H5 and H7 and seasonal H1 antibodies (A/tky/England/647/1977 (H7N7); A/Teal/England 7394-2805/2006 (H5N3); source: European Reference Laboratory for Avian Influenza, Weybridge, UK, and H1N1pdm in-house control strain of FLI, A/Germany/R26/2010 (H1N1pdm)). Frequency and percentages were computed for categorical variables. Median and interquartile range (IQR) were computed for continuous variables. The point prevalence along with the 95% confidence interval (CI) was estimated. Data were analysed with STATA 14.

## Results

### Influenza a prevalence on poultry holdings

Based on questionnaire analyses, most farms (n = 55, 72.4%) had up to 5,000 chickens and the majority (n = 72, 94.7%) kept only layers. Majority of farms (n = 69, 90.8%) reported at least one episode of respiratory infection among the chickens between 3 weeks to 4 months prior to sampling. Nearly all farms (n = 75, 98.7%) retailed their spent layers live, and table eggs at the farm gate. Vaccination against AIV is not practiced in Ghana. AIV was detected in 0.2% (n = 5/2400, 95% CI = 0.19–0.23) of chicken swabs. Viral RNA was detected on 5.3% (n = 4/76) of farms. Four out of five of AIV positive samples were of cloacal origin (Table 1). All positives were detected in layers. The quantitation cycle (Cq) value of all positives ranged from 35 to 38 indicating a very low virus load. The direct subtyping attempt was unsuccessful. Viral isolation attempts failed. AIV antibodies were not detected in any of the 1,200 chicken sera (Table 1).

**Table 1.** Molecular and serological prevalence of AIV detected in chickens.

Sample	Number analysed	No. of positive detected	Prevalence (95% CI)
Cloacal swab	1200	4	0.33 (0.30–0.36)
Tracheal swab	1200	1	0.08 (0.06–0.10)
Serum	1200	0	0

### Influenza a prevalence among chicken farmers

The median age of farmers was 25 years (IQR = 22.0–35.0) and most (n = 74, 72.5%) had worked at the present farm for more than 1 year. Only 2 (2.0%) reported to wear a surgical face mask and none reported to wear gloves when working.

IAV RNA was detected in two swabs from humans. Both were subtyped as H1N1pdm09. Sixteen farmers had IAV antibodies. AIV H5- and H7-specific antibodies were not detected. Antibodies to H1N1pdm09 were detected in 10.8% (11/102) of total sera analyzed (Table 2), and formed 68.8% (11/16) of seropositive samples. All AIV positive farms had a farmer who tested positive to either H1N1pdm09 virus or antibody.

**Table 2.** Molecular and serological prevalence of IAV detected in farmers.

Sample	Number analysed	No. positive	Prevalence (CI)	Influenza sub-/sero-type identified (%)		
				H5	H7	H1N1pdm09
Oropharyngeal swab	102	2	2.0 (1.70–2.22)	0(0)	0(0)	2(2.0)
Serum	102	16	15.7 (8.15–22.0)	0(0)	0(0)	11(10.8)*

*HI titres ≥ 40

## Discussion

The study could not find evidence for endemic circulation of AIV in apparently healthy commercial chickens raised exclusively in-house on farms in the Ashanti region shortly before and during the study period. This is highlighted by the lack of AIV antibodies in any of the chickens examined; following an AIV infection antibodies in layer chickens are expected to be detectable at least 4–6 months after recovery. Thus, past episodes of respiratory disease in layers, as reported by farmers, are most likely unrelated to AIV infections. However, very few cases of active shedding of clinically healthy chickens mostly through faeces were detected. This suggests rare sporadic infection with LPAIV. Subtyping of these viruses was precluded by the very low virus load present in the samples. Previous reports from Ghana likewise did not detect active AIV infection in healthy poultry [18–20] and a low prevalence was reported from Kenya [25].

In several African countries, in contrast, LPAIV alone or in co-infections with other avian pathogens have caused high morbidity, drop in egg production and mortality [26–28]. Interestingly, LPAIV H9N2 in co-infection with infectious bronchitis virus (IBV) caused a significant drop in egg production and high mortality on several layer farms in the Ashanti region a few months after the current study had been finalised. The current study suggests that this virus has not previously circulated in the farms visited but likely was recently introduced into the chicken population, highlighting the consequences of low biosafety measures on farms [29]. Unrestricted moving of AIV-infected live chickens between farms and markets may have played a key role in spreading LPAIV in the country and increase public health risks [30]. The origin of the H9N2 virus later on detected remained unclear but the close phylogenetic relationship to viruses circulating endemically in poultry in several North African countries suggested transboundary i ncursions related to poultry trade [29]. Therefore, raising biosafety standards on poultry farms would be a basic precondition to limit economic losses due to infectious diseases. Controlling trade-related transports of live poultry may further aid in reducing the risk of viral spread. This would be particularly important in case zoonotic AIVs are encountered. Interestingly, the H9N2 viruses causing the reported incursion into Ghana are members of the zoonotic G1 lineage that previously caused human infections in Egypt [31].

Members of the Asian HPAIV H5 lineage with mammalian receptor affinity caused sporadic outbreaks in chicken farms in the Ashanti region, in 2015, 2016 and 2018 [32,33]. The rapid response of the veterinary services of Ghana significantly reduced viral spread and possible contact of farmers with the virus. The absence of H5- and H7-specific antibodies in the farmers despite frequent and long contact to poultry rules out infection with these zoonotic pathogens [7]. In contrast, infections, acute and past, with seasonal human IAV subtype H1N1 was detected. In Nigeria, Cameroon, and Egypt where H5 and H7 antibodies have been detected in poultry workers, the corresponding avian viruses were observed to have circulated for longer periods and affected more poultry holdings, increasing the net exposure risk of poultry workers with possibly infected poultry [15,16,34]. Nevertheless, farmers’ compliance with certain basic biosafety practices were largely poor as noted previously in other parts of the country [18–20] and therefore the risk of exposure to zoonotic AIVs such as LPAIV H9N2 [29] and other non-viral avian pathogens remains high. Co-circulation of IAVs in farmers and their chickens increases the risk of generating reassortants. Regular surveillance of IAVs at the human-animal interface in poultry production for early detection and effective control of these emerging zoonotic and potentially pandemic IAVs would be highly desirable.
